# Normothermic Ex Vivo Machine Perfusion for Liver Transplantation: A Systematic Review of Progress in Humans

**DOI:** 10.3390/jcm12113718

**Published:** 2023-05-28

**Authors:** Charles W. G. Risbey, Carlo Pulitano

**Affiliations:** 1 Department of Surgery, Royal Prince Alfred Hospital, Sydney 2050, Australia; cwgrisbey@gmail.com; 2 Centre for Organ Assessment, Repair & Optimization (COARO), Sydney 2050, Australia; 3 Central Clinical School, The University of Sydney, Sydney 2006, Australia; 4 Department of Transplant Surgery, Royal Prince Alfred Hospital, Sydney 2050, Australia

**Keywords:** normothermic machine perfusion, liver transplantation, static cold storage

## Abstract

Background: Liver transplantation is a lifesaving procedure for patients with end-stage liver disease (ESLD). However, many patients never receive a transplant due to insufficient donor supply. Historically, organs have been preserved using static cold storage (SCS). However, recently, ex vivo normothermic machine perfusion (NMP) has emerged as an alternative technique. This paper aims to investigate the clinical progress of NMP in humans. Methods: Papers evaluating the clinical outcomes of NMP for liver transplantation in humans were included. Lab-based studies, case reports, and papers utilizing animal models were excluded. Literature searches of MEDLINE and SCOPUS were conducted. The revised Cochrane risk-of-bias tool for randomised trials (RoB 2) and the risk of bias in nonrandomised studies for interventions (ROBINS-I) tools were used. Due to the heterogeneity of the included papers, a meta-analysis was unable to be completed. Results: In total, 606 records were identified, with 25 meeting the inclusion criteria; 16 papers evaluated early allograft dysfunction (EAD) with some evidence for lower rates using NMP compared to SCS; 19 papers evaluated patient or graft survival, with no evidence to suggest superior outcomes with either NMP or SCS; 10 papers evaluated utilization of marginal and donor after circulatory death (DCD) grafts, with good evidence to suggest NMP is superior to SCS. Conclusions: There is good evidence to suggest that NMP is safe and that it likely affords clinical advantages to SCS. The weight of evidence supporting NMP is growing, and this review found the strongest evidence in support of NMP to be its capacity to increase the utilization rates of marginal and DCD allografts.

## 1. Introduction

In the United States (USA) alone, more than 1000 patients die whilst waiting for a liver transplant annually, with many more removed from the wait list due to clinical deterioration [1]. A total of 8896 liver transplants were performed in the USA in 2019, an increase of 40.8% from 2009. However, despite the increasing number of liver transplants performed annually, only 49% of wait-listed patients receive a transplant within 12 months [2]. Wait list data shows that after 3 years, 56% of candidates receive a transplant, 11% pass away, 23% are delisted without receiving a transplant, and 9% remain on the waitlist [2]. The reason for the deficit of suitable donor allografts is twofold: firstly, an insufficient supply of donor livers—secondly, graft discard due to viability uncertainty [3].

Historically, donor allografts were preserved for transplantation using static cold storage (SCS), a technique developed in the 1960s which involves cooling the allograft in a preservation solution to reduce cellular metabolism and protect from ischaemic injury [1,4]. Whilst SCS dramatically reduces the metabolic demand of ex situ organs, some anaerobic metabolism persists, leading to the accumulation of metabolic by-products and progressive injury [4]. Graft injury significantly increases once the cold ischaemic time (CIT) passes 7.5 h and in grafts procured following donation after circulatory death (DCD) [4]. Additionally, SCS does not allow dynamic assessment of graft viability, likely leading to significant—and potentially unnecessary—discard rates [4].

Ex vivo machine perfusion is an alternative method of preserving liver allografts for transplantation, with hypothermic (HMP), sub-normothermic (SMP), and normothermic (NMP) variants being described [4]. NMP supplies warmed, oxygenated perfusate to the allograft with the objective of replicating physiological conditions. NMP has generated significant interest recently and may be poised to alter the landscape of liver transplantation through allowing dynamic assessment of graft function, prolonged ex situ graft preservation, increased utilisation of marginal, extended criteria donor (ECD) and DCD grafts, and reduced graft injury compared to the historical standard, SCS.

## 2. Aims

This review aims to synthesise the available evidence investigating the clinical, biochemical, and logistical effects of NMP for liver transplantation in humans and provide a comprehensive overview of the technology.

## 3. Materials and Methods

Papers evaluating the clinical or biochemical outcomes of NMP for liver transplantation in humans were sought. Retrospective and prospective trials were included, as were cohort studies, single-arm studies, and studies using historical-propensity-matched controls. Studies evaluating HMP, SMP, or normothermic regional perfusion (NRP) were excluded. Lab-based studies without transplantation of the graft, studies utilising animal models, studies investigating liver retransplantation, case-control studies, and case reports were excluded.

A comprehensive literature search of MEDLINE and SCOPUS was completed. MEDLINE was searched with the input “human.mp. or Humans/AND liver transplantation.mp. or Liver Transplantation/ AND normothermic.mp AND Perfusion/or Organ Preservation/ or machine perfusion.mp” and SCOPUS with the input “machine AND perfusion AND normothermic AND liver AND transplantation AND human”. No time period or language restrictions were specified. Reference lists of papers identified as meeting the inclusion and exclusion criteria were reviewed to ensure all eligible papers were identified. All retrieved records from MEDLINE and SCOPUS were exported to EndNote 20 for cleaning and appraisal. MEDLINE and SCOPUS were last consulted on 20 October 2022.

Duplicate articles were removed using the de-duplication tool in EndNote 20, and all unique records identified by the literature search were screened individually. Articles clearly not meeting the inclusion criteria based on their title were removed during initial assessment. Abstracts of articles possibly meeting the inclusion and exclusion criteria were assessed and removed only if it could be confidently determined that they did not meet the inclusion criteria. Articles likely to meet the inclusion criteria, and articles unable to be eliminated based on review of their abstracts were retrieved in full. Articles retrieved for review were screened, and individual articles meeting the pre-specified inclusion were ultimately included in this systematic review. Included articles were classified based on their level of evidence using the Australian Government National Health and Medical Research Council (NHMRC) evidence hierarchy, and risk of bias was assessed using the revised Cochrane risk-of-bias tool for randomised trials (RoB 2) for randomised control trials (RCT) or the risk of bias in non-randomised studies for interventions (ROBINS-I) tool for all other included papers [5,6,7]. The NHMRC evidence hierarchy stratifies level of evidence (LOE) based on study design [7]. A systematic review of RCTs is considered LOE-I, an RCT LOE-II, pseudorandomised or comparative studies with concurrent or historical controls LOE-III, and case series LOE-IV [7].

Data were extracted from embedded tables or directly from the body of each individual article. No automated tools were used to extract data from individual reports, nor were any individual study investigators approached to confirm published data. Specific outcomes of interest included early allograft dysfunction (EAD), patient and graft survival, rate of graft discard, utilisation of DCD grafts, biliary complications, and hospital and intensive care unit (ICU) length of stay (LOS). All results that were compatible with each outcome domain were extracted and incorporated in our analysis. Articles which met the inclusion criteria but did not report on these pre-determined clinical outcome domains were considered to have novel clinical endpoints of interest and included in our analysis and conclusions. All extracted data were stored, collated, and organised using Microsoft Excel version 16.73.

To enable systematic synthesis of data, specific outcome domains were grouped, and papers reporting on each domain were included in the analysis. Papers reporting on multiple outcome domains were encompassed in each relevant outcome domain. Microsoft Excel was used to tabulate and organise data for each outcome of interest. This systematic review was completed in accordance with the Preferred Reporting Items for Systematic Reviews and Meta-Analyses (PRISMA) guidelines [8].

## 4. Results

**Figure 1:**
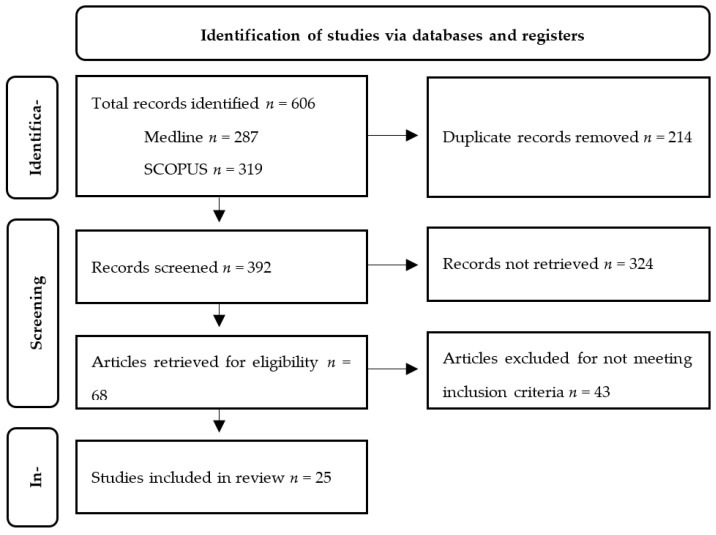
PRISMA flow diagram.

A total of 287 records were retrieved from the search of MEDLINE, and 319 were retrieved from SCOPUS. All 606 records were extracted to EndNote 20, and 214 duplicates were removed. The remaining 392 records were screened individually, with 324 records being discarded and 68 full articles being retrieved for assessment. On review of each full-text article retrieved, 43 were excluded, and a total of 25 records deemed to meet the inclusion criteria (Figure 1). A review of the reference lists of included articles did not identify any articles not previously identified. The characteristics of all 25 papers included in this analysis is shown in Table 1, Table 2 and Table 3. Bias assessment for each paper using the RoB 2 and ROBINS-I tools are outlined in Table 4 and Table 5. Papers appearing to meet the inclusion criteria, however, ultimately excluded include a series of case reports by Zhang et al. [9], a paper by Van Leeuwen et al. which investigated the use of sequential HMP and NMP [10], a paper by Gilbo et al. which investigated coagulation factor accumulation during NMP [11] and a paper by Weissenbacher et al. which investigated the use of perfusate factors and platelets to predict EAD [12].

Five papers investigated the role of NMP in assessing and transplanting previously discarded “orphan grafts” [13,14,15,16,17], three considered ECD allografts only [18,19,20], and two outlined restrictive criteria for an allograft to be considered for NMP [21,22]. Specific NMP techniques also vary between trials, with Guo et al. and Zhang et al. describing novel ischaemia-free liver transplantation (IFLT) techniques [23,24]; and Liu et al. and Selzner et al. describe the use of novel perfusate solutions [25,26]. Additionally, multiple papers use the validated “back-to-base” protocol, which involves transporting and preserving the liver allograft initially using SCS prior to establishing the allograft on NMP at the recipient hospital [14,21,22,27,28]. As such, this significant heterogeneity precluded the pooling of data due to the risk of introducing unnecessary and unacceptable risk of bias and creation of misleading and clinically irrelevant results. As such, this review is qualitative only. 

**Table 1:** Randomised Control Trials.

Author	Year	Design	Location	LOE * [7]	RoB 2 [6]	Device	n	Intervention (NMP)	Control (SCS)
Markmann et al. [29]	2022	Multicentre RCT	USA	II	Low	OCS ^a^	293	n = 151	n = 142
Nasralla et al. [30]	2018	Multicentre RCT	UK	II	Low	OrganOx Metra ^b^	220	n = 120	n = 100
Ghinolfi et al. [31]	2019	Single centre RCT	Italy	II	Low	LiverAssist ^c^	20	n = 10	n = 10

* Level of evidence; ^a^ Organ Care System (Transmedics, Andover, MA, USA); ^b^ OrganOx Metra (OrganOx, Oxford, UK); ^c^ LiverAssist (XVIVO, Goteborg, Sweden)

**Table 2:** Non-Randomised Control Trials.

Author	Year	Design	Location	LOE * [7]	ROBINS-I [5]	Device	n	NMP	SCS
Guo et al. [23]	2021	Prospective, non-randomised control trial	China	III-2	Moderate	LiverAssist ^c^	168	n = 38	n = 130
Chen et al. [18]	2022	Retrospective, non-randomised control trial	China	III-2	Moderate	LiverAssist ^c^	28	n = 14 ^‡^	n = 14
Quintini et al. [13]	2022	Prospective, non-randomised single arm trial	USA	IV	Moderate	Institutional Device	21	n = 21	N/A
Seidita et al. [19]	2022	Retrospective, non-randomised trial	Italy	III-2	Moderate	Not specified	202	n = 19	n = 183
Fodor et al. [21]	2021	Retrospective, non-randomised propensity-score matched trial	Austria	III-3	Moderate	OrganOx Metra ^b^	118	n = 59	n = 59 ^Ω^
MacConmara et al. [32]	2020	Retrospective cohort study	USA	III-2	Serious	Multiple	30,596	n = 228	n = 30,368
Reiling et al. [14]	2020	Prospective, non-randomised single arm trial	Australia	IV	Moderate	OrganOx Metra ^b^	10	n = 10	N/A
Mergental et al. [16]	2020	Prospective, non-randomised propensity-score matched trial	UK	IV	Moderate	OrganOx Metra ^b^	75	n = 31	n = 44 ^Ω^
Cardini et al. [22]	2020	Prospective, non-randomised single arm trial	Austria	IV	Moderate	OrganOx Metra ^b^	34	n = 34	N/A
Zhang et al. [24]	2020	Prospective, non-randomised single arm trial	China	IV	Moderate	LiverAssist ^c^	28	n = 28	N/A
Liu et al. [25]	2020	Prospective, non-randomised propensity-score matched trial	USA	III-3	Moderate	Institutional Device	105	n =21	n = 84 ^Ω^
Watson et al. [17]	2017	Prospective, non-randomised single arm trial	UK	IV	Serious	LiverAssist ^c^	36	n = 12	n = 24 ^†^
Bral et al. [33]	2017	Prospective, non-randomised propensity-score matched trial	Canada	III-3	Moderate	OrganOx Metra ^b^	40	n = 10	n = 30 ^Ω^
Mergental et al. [15]	2016	Prospective, non-randomised single arm pilot series	UK	IV	Serious	LiverAssist ^c^ OrganOx Metra ^b^	6	n = 6	N/A
Selzner et al. [26]	2016	Prospective, non-randomised propensity-score matched trial	Canada	III-3	Moderate	OrganOx Metra ^b^	40	n = 10	n = 30 ^Ω^
Ravikumar et al. [34]	2016	Prospective, non-randomised propensity-score matched trial	UK	III-3	Moderate	OrganOx Metra ^b^	60	n = 20	n = 40 ^Ω^
Jassem et al. [35]	2019	Retrospective, non-randomised propensity-score matched analysis	UK	III-2	Moderate	Not specified	39	n = 12	n = 27 ^Ω^
Gaurav et al. [20]	2022	Retrospective analysis of prospectively collected data	UK	III-2	Moderate	LiverAssist-c OrganOx Metra ^b^	163	n = 67	n = 97
Ionescu et al. [36]	2019	Retrospective, non-randomised propensity-score matched analysis	UK	III-3	Moderate	OrganOx Metra ^b^	144	n = 72	n = 72 ^Ω^

^‡^ n = 7 standard protocol NMP, n = 7 NMP without re-cooling; ^Ω^ matched historical controls; ^†^ comparator cohort.; * level of evidence; ^a^ Organ Care System (Transmedics, Andover, MA, USA); ^b^ OrganOx Metra (OrganOx, Oxford, UK); ^c^ LiverAssist (XVIVO, Goteborg, Sweden)

**Table 3:** Non-randomised control trials investigating novel clinical endpoints.

Author	Year	Design	Location	LOE * [7]	ROBINS-I [5]	Device	n	Intervention	Control
Ceresa et al. [28]	2019	Prospective, non-randomised propensity-score matched trial	UK	III-3	Moderate	OrganOx Metra ^a^	31	n = 31 (SCS/NMP)	n = 104 ^Ω^ (NMP)
Liu et al. [37]	2022	Prospective, non-randomised trial	USA	III-2	Moderate	Institutional Device	15	n = 6 (1 pump NMP system)	n = 9 (2 pump NMP system)
Bral et al. [27]	2019	Prospective, non-randomised trial	Canada	III-2	Moderate	OrganOx Metra ^a^	43	n = 26 (Back-to-base)	N = 17 (Local NMP)

^Ω^ Matched historical controls; * level of evidence; ^a^ OrganOx Metra (OrganOx, Oxford, UK)

**Table 4:** Bias assessment using the RoB 2 assessment tool [6].

Author	Randomisation	Deviation (Assignment)	Deviation (Adhering)	Missing Data	Measurement of Outcomes	Reported Results	Overall
Markmann et al. [29]	Low	Low	Low	Low	Some Concern	Low	Low
Nasralla et al. [30]	Some concern	Low	Low	Low	Low	Low	Low
Ghinolfi et al. [31]	Low	Low	Low	Low	Some concern	Low	Low

**Table 5:** Bias assessment using the ROBINS-I assessment tool [5].

Author	Confounding	Selection	Classification	Deviation	Missing Data	Measurement of Outcomes	Reported Result	Overall
Guo et al. [23]	Moderate	Low	Moderate	Low	Low	Moderate	Low	Low
Chen et al. [18]	Serious	Low	Moderate	Low	Low	Moderate	Moderate	Moderate
Quintini et al. [13]	Serious	Low	Moderate	Low	Low	Moderate	Low	Moderate
Fodor et al. [21]	Serious	Low	Low	Low	Low	Moderate	Moderate	Moderate
MacConmara et al. [32]	Critical	Low	Moderate	Low	Moderate	Serious	Low	Serious
Seidita et al. [19]	Critical	Low	Moderate	Low	Low	Moderate	Low	Moderate
Reiling et al. [14]	Serious	Low	Moderate	Low	Low	Moderate	Low	Moderate
Mergental et al. [16]	Serious	Moderate	Moderate	Low	Low	Moderate	Low	Moderate
Cardini et al. [22]	Serious	Moderate	Moderate	Low	Low	Moderate	Moderate	Moderate
Zhang et al. [24]	Critical	Low	Moderate	Low	Low	Moderate	Moderate	Moderate
Liu et al. [25]	Serious	Low	Moderate	Low	Low	Moderate	Moderate	Moderate
Liu et al. [37]	Critical	Moderate	Moderate	Low	Low	Moderate	Low	Moderate
Ceresa et al. [28]	Serious	Moderate	Moderate	Low	Low	Moderate	Low	Moderate
Ionescu et al. [36]	Serious	Moderate	Moderate	Low	Low	Moderate	Moderate	Moderate
Bral et al. [27]	Serious	Moderate	Moderate	Low	Low	Low	Moderate	Moderate
Watson et al. [38]	Critical	Moderate	Moderate	Moderate	Moderate	Moderate	Moderate	Serious
Bral et al. [33]	Serious	Moderate	Moderate	Low	Low	Moderate	Moderate	Moderate
Mergental et al. [15]	Critical	Moderate	Moderate	Moderate	Low	Low	Moderate	Serious
Selzner et al. [26]	Critical	Moderate	Moderate	Low	Low	Moderate	Moderate	Moderate
Ravikumar et al. [34]	Critical	Moderate	Moderate	Moderate	Low	Moderate	Moderate	Moderate
Jassem et al. [35]	Critical	Moderate	Moderate	Moderate	Low	Low	Moderate	Moderate
Gaurav et al. [20]	Serious	Moderate	Moderate	Low	Low	Moderate	Moderate	Moderate

### 4.1. Early Allograft Dysfunction

All articles evaluating the rate of EAD utilise the definition of EAD proposed and validated by Olthoff et al. [39]. Sixteen papers reported rates of EAD, with all three RCTs including EAD as an endpoint (Table 6). Large RCTs by both Markman et al. and Nasralla et al. describe a standard NMP protocol, and both report significantly lower rates of EAD following allograft preservation using NMP compared to SCS (18% cf. 31.5%, *p* = 0.001 and 10.1% cf. 29.9%, *p* < 0.01, respectively) [29,30]. The baseline characteristics of donor grafts allocated to each arm in both trials were relatively well matched. However, the NMP arm in the Markmann et al. trial did have a higher rate of DCD, 19% versus 8%, although it is not clear whether this reached statistical significance [29]. Combined, these two trials encompass a total of 513 patients, providing a high level of evidence (NHMRC level II) that NMP reduces the rate of EAD. The third RCT by Ghinolfi et al. is a pilot trial with a total of 20 patients, precluding it from recording statistically significant results [31].

The prospective, non-randomised trial by Guo et al. describes a novel IFLT technique, which avoids cold ischaemia time (CIT) during procurement and implantation [23]. The IFLT technique is not standard protocol and is only possible using DBD allografts, meaning the results of Guo et al. are not necessarily translatable to the wider use of NMP. Regardless, their results show a significant reduction of EAD with IFLT compared to SCS (absolute risk difference 44.8%, 95% CI: 33.6–55.9%, *p* < 0.001). Chen et al. also describe a novel surgical technique which avoids a second period of CIT during implantation [18]. Their retrospective analysis contains three arms: NMP without re-cooling (n = 7), standard NMP with cooling during procurement and implantation (n = 7) and SCS (n = 14). The rate of EAD was not significant between all three arms (*p* = 0.089), however, subgroup analysis demonstrated NMP without re-cooling significantly reduced rates of EAD compared to SCS (0% cf. 50%, *p* = 0.022) [18]. Liu et al. also describe another novel NMP technique utilising a fresh frozen plasma (FFP)-based perfusate as opposed to Gelofusine or Steen solution used in many other NMP trials [25]. The authors report a significant reduction in EAD following NMP when compared to SCS (19 cf. 56%, *p* = 0.02). However, similarly to Guo et al. and Chen et al., they describe a novel technique which may not be applicable to the wider use of NMP technology.

The prospective, single-arm trial with contemporary matched controls by Mergental et al. was designed to assess the viability of NMP to rescue previously discarded orphan grafts [16]. All 22 NMP-transplanted livers had previously been rejected by all transplant centres across the UK, and many were reported to appear macroscopically suboptimal. Nevertheless, the authors report a significantly higher rate of EAD following transplantation of orphan grafts using NMP when compared to a cohort of matched SCS controls (odds ratio 5.6, 95% CI: 1.1–27.8, *p* = 0.034). Three propensity-matched trials by Fodor et al., Bral et al., and Ravikumar et al. report no significant difference between rates of EAD for livers transplanted with NMP when compared to SCS [21,33,34]. All three provided a relatively low level of evidence (NHMRC level III-3) and had a moderate risk of bias according to the ROBINS-I tool. However, they encompassed a total of 89 NMP cases and 129 historical propensity-matched controls. The reported rate of EAD following NMP of 55.5% by Bral et al. is the highest of any study, which is notable considering that multiple papers report rates of 5% or below [15,18,23,24]. This brings into question whether their results are affected by undescribed confounding factors. The remaining papers by Quintini et al., Reiling et al., Cardini et al., Zhang et al., Mergental et al., and Gaurav et al. all report rates of EAD between 0 and 50% following NMP. However, all are single-arm studies with no comparators [13,14,15,20,22,24].

Overall, the true rate of EAD following NMP when compared to SCS is difficult to ascertain given the heterogeneity of evidence and the inability to complete a pooled analysis due to trials utilising novel NMP techniques or demonstrably different patient cohorts. Despite this, the two largest trials encompassing the highest quality of evidence both show a significant reduction in EAD using NMP when compared to SCS, which would lend weight to the view that NMP is superior in this regard.

### 4.2. Graft and Patient Survival

19 papers investigate graft and patient survival; however, no paper reports a significant difference for either endpoint (Table 7). This lack of significance is reflected in the retrospective data linkage study completed by MacConmara et al., which linked data from the UNOS database and the Social Security Death Master File [32]. This paper encompasses a total of 30,596 cases from 2016 through 2019, including 220 successful NMP transplantations and 26,330 successful SCS transplantations. The authors used propensity scoring to match individual NMP with SCS transplantations at a ratio of 1:10, and their findings demonstrate a trend toward increased graft and patient survival at 1 year for recipients of SCS allografts, although not significant, with *p* = 0.11 and *p* = 0.20, respectively. However, a lack of detail regarding the propensity score matching process and baseline donor characteristics mean it is not possible to rule out confounding or selection bias, especially given baseline characteristics for all NMP liver transplants recipients were significantly more likely to be older (47.7 cf. 39.5 years, *p* < 0.0001), have a higher BMI (29.8 cf. 27.7 kg/m^2^, *p* < 0.0001), originate from DCD (18 cf. 7%, *p* < 0.0001) and ECD (18.2 cf. 6.9%, *p* < 0.0001) when compared to SCS [32].

Most early studies were designed and powered to demonstrate the safety of NMP, not its superiority, given it was a new technology competing with a proven reliable, safe, and well-understood allograft preservation method. Available evidence demonstrates the safety of NMP when considering both patient and graft survival, as outlined by all three RCTs, which report patient and graft survival at 12 months to be non-inferior to SCS [28,29,30]. There is no discernible trend among the trials, and Markmann et al. report a statistically significant non-inferiority (*p* < 0.001) patient survival for NMP compared to SCS at both 1 and 12 months [29]. The next largest trial, authored by Guo et al., also reports no significant difference in graft or patient survival at 12 months with their IFLT technique compared to SCS (89.5 cf. 81.5%, *p* = 0.326 and 92.1 cf. 82.3%, *p* = 0.142 respectively) [23]. Guo et al. demonstrate patient and graft survival following their IFLT technique to be similar to the survival rates reported in the NMP arm of the RCTs by Markmann et al. and Nasralla et al. However, patient and graft survival in their SCS arm is 10–15% lower at 12 months. The significance of this is unclear.

The trial with the longest period of follow-up was conducted by Seidita at al., who completed a retrospective analysis of 202 liver transplants with a 3-year follow-up period [19]. At 3 years, they found no significant difference in graft or patient survival. A small prospective, non-randomised trial encompassing 10 NMP and 30 matched control SCS transplants by Bral et al. reported that 8 of 10 allografts survived to 6 months following NMP preservation compared to 100% graft survival of the matched SCS recipients on an intent-to-treat basis (*p* = 0.06) [33]. However, one of the NMP grafts experienced a portal venous twist on the OrganOx Metra device and was discarded prior to implantation, meaning results from an as-treated cohort were more uniform between groups [33].

### 4.3. Biliary Complications

Biliary complications were an endpoint for 13 papers (Table 8). Specific endpoints evaluated included ischaemic cholangiopathy (IC), non-anastomotic strictures (NAS), and anastomotic strictures (AS). Markmann et al. reported no difference in the rate of AS between NMP and SCS (11.1 cf. 11.6%, *p* = 1.0). However, they did report a significant reduction in the rate of IC with NMP (2.6 cf. 9.6%, *p* = 0.002) [29]. Conversely, Nasralla et al. reported no difference in the rates of AS, NAS, or IC between NMP and SCS as seen on MRCP [30]. It should be noted, however, that Nasralla et al. reported rates of biliary complications seen on MRCP rather than clinically significant complications. Ghinolfi et al. reported one biliary complication in the NMP arm of their trial. However, as their RCT was comprised of 10 patients in each arm, this was not significant [31].

Fodor et al. reported no overall difference in rates of biliary complications between NMP and SCS (50.8 cf. 49.2%, *p* = 0.854). However, sub-analysis showed a significant reduction in IC with NMP (3.4 cf. 13.6%, *p* = 0.047) [21]. This finding is in line with the findings of Markman et al., who also found the risk of IC to be reduced with NMP. However, the study protocol of Fodor et al. did have restrictive donor-related, recipient-related, and logistic related requirements for NMP preservation [21,29]. Guo et al. also reported a trend towards lower rates of biliary complications following their IFLT technique. However, their data did not reach statistical significance [23].

Conversely, Mergental et al. and Chen et al. reported a trend towards an increased rate of biliary complications associated with NMP. However, neither reported statistically significant results [16,18]. Mergental et al. included only orphan grafts in their NMP cohort and utilised historical matched controls for their SCS comparator group, meaning the quality of evidence is low (NHMRC IV) [16]. Chen et al. designed their study with 3 arms, incorporating a modified NMP protocol without re-cooling prior to transplantation, reducing the clinical applicability of their results [18]. Overall, There is little evidence to suggest that NMP reduces the overall rate of biliary complications. However, there is some evidence that NMP reduces the rate of IC, as demonstrated by the significant results reported by Markmann et al. and Fodor et al. [21,29].

### 4.4. Rate of Allograft Discard and Utilisation of DCD

Both large RCTs provide good evidence that NMP increases the utilisation of marginal or ECD liver allografts [29,30]. Markmann et al. showed a significantly higher rate of DCD utilisation amongst the NMP arm (51% cf. 26%, *p* = 0.007), whilst Nasralla et al. demonstrated a significantly reduced rate of allograft discard in the NMP group (11.7% cf. 24%, *p* = 0.008) [29,30]. The large retrospective analysis of the UNOS database completed by MacConmara et al. also provides compelling evidence for higher rates of DCD graft utilisation (18% cf. 6.9% *p* < 0.001) and a lower rate of overall graft discard (3.5% cf. 13.3%, *p* < 0.001) with NMP amongst their cohort of 30,596 cases [32]. It should be noted, however, that viability criteria for each allograft perfused using NMP are often poorly described and lacks uniformity across papers.

All NMP cases included in the trials by Quintini et al., Reiling et al., and those in both papers penned by Mergental et al. were discarded orphan allografts that had been previously rejected for transplantation [11,12,13,14]. Across all four papers, a total of 68 orphan grafts were assessed using NMP, with quoted graft rescue rates of 71.5–100%. Each paper was a single-arm non-randomised trial, with the objective to show proof of concept that NMP is an effective means to assess and, ultimately, successfully transplant orphan grafts. These findings demonstrate promise that NMP can reduce the rate of organ discard. However, they do not provide insights into the overall rate of allograft discard using NMP compared to SCS (Table 9).

### 4.5. Length of Stay

The RCT authored by Nasralla et al. encompasses a total of 220 transplantations. However, it failed to detect a significant difference for ICU or hospital LOS between NMP and SCS [30]. The small RCT by Ghinolfi et al. showed a trend towards a longer hospital LOS following NMP graft preservation, however, their results do not reach statistical significance (17 cf. 12 days, *p* = 0.119). Notably, the RCT by Markman et al. does not report on ICU or hospital LOS [29].

Guo et al. reported that IFLT is associated with a significantly shorter ICU LOS (1.48 cf. 1.81 days, *p* = 0.006) but reported no reduction in overall hospital LOS compared to SCS. Conversely, the small, non-randomised, prospective-propensity-score-matched trial by Bral et al. reported NMP allograft preservation to be associated with significantly longer ICU LOS (median 16 cf. 4 days, *p* = 0.004) [33]. Of note, the reported median ICU LOS of 16 days (range 2–65 days) by Bral et al. is considerably longer than the next-highest reported ICU LOS by Nasralla et al. of 4 days for NMP [30,33]. The significance of this is unknown. However, as their results diverge significantly from the reported ICU LOS of all other studies, it is possible they are affected by the presence of institutional, technical, or patient-related confounding factors. All other studies report no significant difference in ICU LOS between NMP and SCS (Table 10).

Fodor et al. reported a significantly shorter total hospital LOS following the transplantation of allografts preserved using NMP (median 17 cf. 23 days, *p* = 0.006) [21]. This is in contrast to Bral et al., who reported that NMP is associated with a longer hospital LOS (median 45 cf. 25 days, *p* = 0.01) [33]. Similar to their data regarding ICU LOS, the median hospital LOS reported by Bral et al. diverges from the other 12 papers reporting hospital LOS. The next-highest hospital LOS is reported by Guo et al., who found that patients remained in hospital for a mean 19.5 days following transplantation using NMP, again raising the possibility that their results are influenced by confounding bias. All other papers failed to find a statistically significant difference in duration of total hospital admission between each group. Overall, there is no strong evidence to suggest that either NMP or SCS is associated with shorter ICU or hospital admissions.

### 4.6. Intraoperative Coagulation Profiles and Blood Product Use

Ionescu et al. showed that preserving liver allografts using NMP is associated with significantly improved intraoperative coagulation profiles and reduced requirement for intraoperative platelet transfusion [36]. Their retrospective, non-randomised propensity-matched trial included a total of 72 NMP transplants matched 1:1 with propensity score controls [36]. Their results found NMP is associated with significantly improved intraoperative thromboelastography (TEG) profiles with reduced time to reach a clot strength of 20 mm (K-time; *p* = 0.010), increased rate of clot formation (a-angle; *p* = 0.002), increased clot lysis time (*p* = 0.004) and increased maximum clot strength (MA; *p* = 0.044) [36]. Clinically this resulted in a significant reduction in intraoperative platelet transfusion requirement with NMP (34 cf. 64%, *p* = 0.001), however, no change to the requirement of FFP (*p* = 0.070), packed red cells (*p* = 0.655) or cryoprecipitate (*p* = 1.00) [36].

## 5. Discussion

Whilst a total of 25 papers were identified to meet the inclusion criteria for this review, there was significant heterogeneity in regard to study design, donor characteristics, surgical technique, and transplantation protocols between papers. Additionally, the lack of standardised viability criteria between trials obscures the interpretation of results and perpetuates heterogeneity between trials. Many papers use common perfusion endpoints, such as lactate clearance, perfusate pH, bile production, vascular flows, and macroscopic inspection to assess organ viability. However, cut-off values and the combination of parameters assessed are highly variable between papers. This heterogeneity prevented pooled analyses due to the unacceptable risk of introducing selection, confounding, and technical biases. As such, only qualitative analyses were possible, limiting the generalisability and certainty of our findings. A narrower focus and more restrictive inclusion criteria to ensure included studies are sufficiently homogenous would be required to complete a high-quality meta-analysis on specific outcome domains.

The highest-quality evidence for NMP is provided by two large RCTs by Markmann et al. and Nasralla et al. [29,30]. Both of these trials directly compare NMP and SCS. However, Nasralla et al. employed the OrganOx Metra (OrganOx Ltd., UK) device, and Markmann et al. employed the Organ Care System (Transmedics, USA), both single-pump, commercially available NMP circuits. Both trials included standard criteria donor (SCD) allografts and described a standard NMP protocol with a period of CIT prior to establishing each donor allograft on NMP and again prior to transplantation. Both trials suggest that significant benefits are afforded by NMP, with statistically significant reductions in EAD, biliary complications, and rate of allograft discard in addition to higher rates of DCD graft utilisation being reported by one or both trials. Neither trial, however, showed improvements to patient or graft survival with NMP, but Markman et al. did report that NMP was non-inferior to SCS (non-inferiority *p* < 0.001). As such, these RCTs—encompassing a total of 513 patients—provide high-level evidence that NMP is—at minimum—non-inferior and likely associated with clinical benefits compared to SCS.

Whilst both RCTs were based on similar study designs, each of the largest non-randomised control trials differ significantly in their design. Guo et al. employed an IFLT technique, which eliminates CIT; Seidita et al. incorporated only ECD allografts; Gaurav et al. incorporated only DCD allografts; and Fodor et al. stipulated restrictive inclusion criteria based on high-risk donor, recipient, or logistic factors [17,18,19,21]. As such, results from these papers are less generalisable. However, like Markmann at al. and Nastalla et al., all suggest NMP is at least non-inferior to SCS. In particular, the single-centre, non-randomised prospective trial by Guo et al., which encompassed a total of 168 patients, suggests significant benefits associated with their IFLT technique [23]. Their reported reduction in EAD (5.3% cf. 50.0%, *p* < 0.001) and ICU LOS (1.48 cf. 1.81 days, *p* = 0.006) provide evidence to suggest that their IFLT technique has benefit. However, teasing out the relative effect of eliminating CIT versus the benefits of NMP is not possible without further investigation.

Whilst reductions in EAD and reduced peak post-operative transaminase levels have been reported to be surrogate markers for graft function [40,41], the long-term benefits of NMP are yet to be proven, and it is important to note that no paper has shown a survival benefit for NMP. Guo et al. presented the most compelling data in favour of NMP being associated with a survival benefit; however, their data still fail to reach significance (92.1% cf. 82.3% 12-month survival, *p* = 0.142) [23]. Regardless, the inability to demonstrate a survival benefit afforded by NMP may not be significant concern given some papers were designed to demonstrate survival non-inferiority. Ultimately, there is good evidence to suggest NMP is associated with non-inferior patient and graft survival rates when compared to SCS. However, further large, sufficiently powered papers will be required to better characterise any long-term clinical benefits of NMP.

Despite a lack of evidence to suggest improved survival outcomes, there is good evidence that NMP can reduce allograft discard rates and increase DCD graft utilisation. The ability to assess donor allograft viability using SCS is limited. However, NMP allows dynamic assessment of biochemical and synthetic function, vascular characteristics, and bile production, which greatly enhance graft evaluation [42,43]. Both studies which reported rates of graft discard and DCD utilisation demonstrated benefits associated with NMP, however, the paper by MacConmara et al. is particularly compelling, as their results include all liver transplants completed in the US over the 3-year period from 2016 through 2019 [29,30,32]. Whilst there are inevitable issues and risk of bias with their retrospective analysis, their extraction of data from the UNOS database provides credible evidence that the benefits of NMP for improved allograft utilisation seen in trial settings translate to clinical practice. Furthermore, multiple papers demonstrated that NMP can facilitate successful transplantation of allografts which had previously been rejected for clinical use. The findings of Quintini et al., Reiling et al., and Mergental et al. that NMP can lead to a 71–100% rescue rate of previously discarded orphan grafts provide compelling evidence that NMP has the potential to expand the donor pool [13,14,15,16]. This finding is possibly the most persuasive endorsement for NMP, as thousands of potentially viable allografts are discarded annually, and technological advancements that facilitate the successful transplantation of these organs have the potential to reduce wait-list times and prevent unnecessary patient mortality.

A significant weakness of the overall quality of evidence for NMP is the large number of studies with small sample sizes and the reliance on propensity-matched controls. In total, 12 of the 22 papers directly comparing NMP to SCS have 21 or fewer patients in the NMP arm of each trial; 8 papers rely on propensity-matched controls, and 6 are single-arm trials with no comparator cohort. This lack of sufficiently powered trials with contemporary controls not only impacts the ability to detect subtle effects of NMP but also introduces significant risk of bias. This issue may have been mitigated through the pooling of data; however, this was not possible due to the heterogeneity of the study design and patient cohorts. Additionally, papers without comparator cohorts provide useful insights into the increasing use of NMP worldwide. However, they provide little evidence to support the continued expansion of the technology.

An important consideration is that all studies included in this review describe a short-term model of NMP. Each paper utilised NMP for less than 24 h prior to transplantation, with the majority perfusing each liver with NMP for only a small number of hours. This is notable given that recent technological advancements allow prolonged ex vivo perfusion of livers using NMP [44]. Our group has reported successful perfusion of human livers for up to 13 days using a modified long-term NMP system, proving an important evolution of the technology [45,46,47]. Prolonged perfusion allows close observation, dynamic assessment, therapeutic intervention, and possible regeneration of each graft, possibly broadening the scope of benefit. Recently, Clavien et al. successfully transplanted a liver after 3 days of NMP perfusion, demonstrating the viability of this concept [48]. As such, it is plausible that investigating clinical and biochemical outcomes following a short period of NMP falls short of identifying many clinical benefits associated with the technology, which may only be associated with the long-term perfusion of grafts.

NMP has also been described as an effective preservation technique for other thoracic and abdominal organs for transplantation. Its use in kidney transplantation is expanding, and emerging evidence suggests that it can facilitate improved viability testing and reduced organ discard rates, mirroring the findings of this review [49]. NMP has also been shown to be safe when used for lung preservation and to be able to extend total preservation time [50,51]. The use of NMP for preservation of pancreas grafts is limited, however, efforts are underway to explore possible techniques to use NMP in this context [52]. Overall, the benefits of NMP appear to not be limited to liver transplantation. Rather, they may play an expanding role in the preservation and assessment of other thoracic and abdominal organs for transplantation.

Overall, there is a significant need for further large, well designed, and generalisable trials evaluating the long-term clinical effects of NMP. An abundance of evidence demonstrates the viability of NMP technology, but data on long-term benefit are lacking. In particular, future studies should be designed to investigate clinically relevant endpoints, such as surgical or graft complication rates, graft and patient survival, or rates of re-transplantation. Until there is compelling evidence that NMP is associated with significant and durable patient benefit, the economic cost, technological challenges, and novel skills required to use NMP will likely impair its uptake into everyday clinical practice.

## 6. Conclusions

The evidence base for NMP is advancing rapidly, with all papers identified to meet the inclusion criteria for this review being published more recently than 2016. Whilst no paper has been able to show a survival benefit for NMP, the lack of well-designed, sufficiently powered papers with follow-up beyond 12 months means it is not possible to draw robust conclusions to this endpoint. Despite this, there is some evidence to suggest that NMP leads to lower risk of EAD and biliary complications compared to SCS, and there is high-quality evidence to suggest that NMP can facilitate the increased utilisation of ECD and orphan grafts. Overall, it is evident that NMP has the potential to revolutionise liver transplantation, however, further high-quality papers, possibly with a focus on long-term NMP protocols, are required to demonstrate its superiority to SCS.

## Figures and Tables

**Table 6:** Rate of EAD.

Author	NMP (%)	SCS (%)	*p*
Markmann et al. [29]	18	31.5	*p* = 0.01
Nasralla et al. [30]	10.1	29.9	*p* < 0.001
Ghinolfi et al. [31]	20	10	*p* = 1.00
Guo et al. [23]	5.3	50.0	*p* < 0.001
Chen et al. [18]	mNMP ^Ω^: 0 NMP: 28.5	50	*p* = 0.089 ^‡^ *p* = 0.022 ^†^
Quintini et al. [13]	46.6	N/A	N/A
Fodor et al. [21]	32	34	*p* = 0.794
Reiling et al. [14]	50	N/A	N/A
Mergental et al. [16]	32	9.1	*p* = 0.034
Cardini et al. [22]	20	N/A	N/A
Zhang et al. [24]	3.6	N/A	N/A
Liu et al. [25]	19	46	*p* = 0.02
Bral et al. [33]	55.5	29.6	*p* = 0.23
Mergental et al. [15]	0	N/A	N/A
Ravikumar et al. [34]	15	22.5	*p* = 0.734
Gaurav et al. [20]	11	21	N/A

^Ω^ Modified NMP protocol without re-cooling; ^‡^ analysis of NMP vs. mNMP ^Ω^ vs. SCS; ^†^ analysis of mNMP ^Ω^ vs. SCS.

**Table 7:** Rate of patient and graft survival.

Author	Graft Survival (%)			Patient Survival (%)		
	NMP	SCS	*p*	NMP	SCS	*p*
Markmann et al. [29]				99.3 (1 mth) 94 (12 mths)	99.3 (1 mth) 93.7 (12 mths)	Noninferiority *p* < 0.001
Nasralla et al. [30]	95 (12 mths)	96 (12 mths)	*p* = 0.707	96 (12 mths)	97 (12 mths)	*p* = 0.671
Ghinolfi et al. [31]	90 (12 mths)	100 (12 mths)	*p* = 1.000	100 (12 mths)	90 (12 mths)	*p* = 1.000
Guo et al. [23]	97.4 (1 mth) 89.5 (12 mths)	90.0 (1 mth) 81.5 (12 mths)	*p* = 0.195 *p* = 0.326	97.4 (1 mth) 92.1 (12 mths)	90.8 (1 mth) 82.3 (12 mths)	*p* = 0.302 *p* = 0.142
Chen et al. [18]				mNMP ^Ω^ 85.8 (30 dy) NMP 100 (30 dy) mNMP ^Ω^ 85.8 (90 dy) NMP 100 (90 dy)	85.8 (30 dy) 85.8 (90 dy)	*p* = 0.571 *p* = 0.571
Fodor et al. [21]	97 (1 mth) 89 (3 mths) 81 (1 yr)	98 (1 mth) 93 (3 mths) 82 (1 yr)	*p* = 0.347	95 (1 mth) 89 (3 mths) 81 (1 yr)	95 (1 mth) 91 (3 mths) 79 (1 yr)	*p* = 0.784
MacConmara et al. [32]	N/A	N/A	*p* = 0.11	N/A	N/A	*p* = 0.20
Seidita et al. [19]	88 (1 mth) 88 (3 mths) 88 (6 mths) 88 (1 yr) 76 (3 yrs)	98 (1 mth) 95 (3 mths) 92 (6 mths) 90 (1 yr) 80 (3 yrs)	*p* = 0.577	94 (1 mth) 94 (3 mths) 94 (6 mths) 94 (1 yr) 82 (3 yrs)	98 (1 mth) 96 (3 mths) 92 (6 mths) 90 (1 yr) 80 (3 yrs)	*p* = 0.697
Reiling et al. [14]	100 (3 mths) 100 (6 mths) 100 (1 yr)	N/A	N/A	100 (3 mths) 100 (6 mths) 100 (1 yr)	N/A	N/A
Mergental et al. [16]	100 (3 mth) 86.4 (1 yr)	93.2 (3 mth) 86.4 (1 yr)	*p* = 0.545 *p* = 1.00	100 (3 mth) 100 (1 yr)	100 (3 mth) 95.5 (1 yr)	*p* = 1.00 *p* = 0.55
Cardini et al. [22]	88 (20 mths)	N/A	N/A	88 (20 mths)	N/A	N/A
Liu et al. [25]	100 (6 mth) 95.2 (12 mth)	N/A	N/A	100 (6 mth) 95.2 (12 mth)	N/A	N/A
Watson et al. [17]	83 (9 mths)	88 (9 mths)	N/A	92 (9 mths)	96 (9 mths)	N/A
Bral et al. [33]	90 (1 mth) 80 (6 mth)	100 (1 mth) 100 (1 mth)	*p* = 0.25 *p* = 0.06	100 (1 mth) 89 (6 mths)	100 (1 mth) 100 (6 mths)	N/A *p* = 0.25
Mergental et al. [15]	100 (3 mths)	N/A	N/A	100 (3 mths)	N/A	N/A
Selzner et al. [26]	100 (3 mths)	100 (3 mths)	N/A	100 (3 mths)	100 (3 mths)	N/A
Ravikumar et al. [34]	100 (1 mth)	97.5 (1 mth)	*p* = 1.00	100 (1 mth) 100 (6 mths)	97.5 (1 mth) 97.5 (6 mths)	*p* = 1.00 *p* = 1.00
Jassem et al. [35]	100	100	*p* = 1.00	91.7	100	N/A
Gaurav et al. [20]	91 (6 mths)	91 (6 mths)	N/A	94 (6 mths)	96 (6 mths)	*p* = 0.90

^Ω^ Modified NMP protocol without re-cooling.

**Table 8:** Biliary complications.

Author	NMP (%)	SCS (%)	*p*
Markmann et al. [29]	11.1 ˆ 2.6 ^‡^	11.6 ˆ 9.6 ^‡^	*p* = 1.0 *p* = 0.02
Nasralla et al. [30]	8.6 * 43.2 ˆ 0.8 ^‡^	10.8 * 45.9 ˆ 1 ^‡^	N/A
Ghinolfi et al. [31]	10	0	*p* = 1.000
Guo et al. [23]	10.5 0 * 7.9 ˆ	18.5 3.8 * 12.3 ˆ	*p* = 0.326
Chen et al. [18]	mNMP ^Ω^ 0 ˆ NMP 7.1 ˆ	SCS 0ˆ	*p* = 0.211
Quintini et al. [13]	6.7 ^‡^	N/A	N/A
Fodor et al. [21]	50.8 35.6 * 8.5 ˆ 3.4 ^‡^	49.2 39.0 * 16.9 ˆ 13.6 ^‡^	*p* = 0.854 *p* = 0.703 *p* = 0.167 *p* = 0.047
Reiling et al. [14]	20	N/A	N/A
Mergental et al. [16]	18.2 * 9.1 ˆ	2.3 * 6.8 ˆ	*p* = 0.063 *p* = 0.725
Cardini et al. [22]	36	N/A	N/A
Watson et al. [38]	27 ^‡^	29 ^‡^	N/A
Bral et al. [33]	0	14.8	*p* = 0.55
Gaurav et al. [20]	37	42	N/A

^Ω^ Modified NMP protocol without re-cooling; * non-anastomotic stricture; ˆ Anastomotic stricture; ^‡^ Ischaemic cholangiopathy.

**Table 9:** Rate of allograft discard and DCD utilisation.

Author	Rate of Discard (%)			Utilisation of DCD (%)		
	NMP	SCS	*p*	NMP	SCS	*p*
Markmann et al. [29]				51	26	*p* = 0.007
Nasralla et al. [30]	11.7	24.1	*p* = 0.008	54	35	N/A
Quintini et al. [13]	28.5 ^‡^	N/A	N/A			
MacConmara et al. [32]	3.5	13.3	*p* < 0.001	18.2	6.9	*p* < 0.001
Seidita et al. [19]	10.5	N/A	N/A			
Reiling et al. [14]	0 ^‡^	N/A	N/A	100 ^‡^	N/A	N/A
Mergental et al. [16]	29 ^‡^	N/A	N/A	70.6	N/A	N/A
Cardini et al. [22]	26.5	N/A	N/A	100	N/A	N/A
Mergental et al. [15]	16.7 ^‡^	N/A	N/A			
Selzner et al. [26]	16.7	N/A	N/A			

^‡^ Discarded orphan grafts.

**Table 10:** Hospital and ICU LOS.

Author	ICU LOS (Days)			Hospital LOS (Days)		
	NMP	SCS	*p*	NMP	SCS	*p*
Nasralla et al. [30]	4	4	*p* = 0.339	15	15	*p* = 0.926
Ghinolfi et al. [31]				17	12	*p* = 0.119
Guo et al. [23]	1.48	1.81	*p* = 0.006	19.5	21.5	*p* = 0.795
Fodor et al. [21]	3	4	*p* = 0.638	17	23	*p* = 0.006
Reiling et al. [14]	1.5	N/A	N/A	11.5	N/A	N/A
Mergental et al. [16]	3.5	2	*p* = 0.566	10	9	*p* = 0.822
Liu et al. [25]	2.5	2.7	*p* = 0.27	13.4	15.7	*p* = 0.49
Bral et al. [33]	16	4	*p* = 0.004	45	25	*p* = 0.01
Mergental et al. [15]	3.8	N/A	N/A	10	N/A	N/A
Selzner et al. [26]	1	2	*p* = 0.54	11	13	0.23
Ravikumar et al. [34]	3	3	*p* = 0.459	12	14	*p* = 0.100
Jassem et al. [35]	3	5	NS •			
Gaurav et al. [20]	2	2	N/A	19	18	N/A

• Not significant.

## Data Availability

All data are presented within the manuscript.

## References

[1] Tchilikidi K.Y. (2019). Liver graft preservation methods during cold ischemia phase and normothermic machine perfusion. World J. Gastrointest. Surg..

[2] Kwong A.J., Kim W.R., Lake J.R., Smith J.M., Schladt D.P., Skeans M.A., Noreen S.M., Foutz J., Booker S.E., Cafarella M. (2021). OPTN/SRTR 2019 Annual Data Report: Liver. Am. J. Transplant..

[3] Akateh C., Beal E.W., Whitson B.A., Black S.M. (2018). Normothermic Ex-vivo Liver Perfusion and the Clinical Implications for Liver Transplantation. J. Clin. Transl. Hepatol..

[4] Aufhauser D.D., Foley D.P. (2021). Beyond Ice and the Cooler: Machine Perfusion Strategies in Liver Transplantation. Clin. Liver Dis..

[5] Sterne J.A.C., Hernán M.A., Reeves B.C., Savović J., Berkman N.D., Viswanathan M., Henry D., Altman D.G., Ansari M.T., Boutron I. (2016). ROBINS-I: A tool for assessing risk of bias in non-randomised studies of interventions. BMJ.

[6] Sterne J.A.C., Savović J., Page M.J., Elbers R.G., Blencowe N.S., Boutron I., Cates C.J., Cheng H.Y., Corbett M.S., Eldridge S.M. (2019). RoB 2: A revised tool for assessing risk of bias in randomised trials. BMJ.

[7] Merlin T., Weston A., Tooher R., Middleton P., Salisbury J., Coleman K., Norris S., Grimmer-Somers K., Hillier S., Council NHaMR (2009). NHMRC Levels of Evidence and Grades for Recommendations for Developers of Guidelines.

[8] Page M.J., McKenzie J.E., Bossuyt P.M., Boutron I., Hoffmann T.C., Mulrow C.D., Shamseer L., Tetzlaff J.M., Akl E.A., Brennan S.E. (2021). The PRISMA 2020 statement: An updated guideline for reporting systematic reviews. Rev. Esp. Cardiol. (Engl. Ed.).

[9] Zhang Z., Ju W., Tang Y., Wang L., Zhu C., Gao N., Zhao Q., Huang S., Wang D., Yang L. (2020). First Preliminary Experience with Preservation of Liver Grafts from Extended-Criteria Donors by Normothermic Machine Perfusion in Asia. Ann. Transplant..

[10] van Leeuwen O.B., Bodewes S.B., Lantinga V.A., Haring M.P., Thorne A.M., Brüggenwirth I.M., Berg A.P.V.D., de Boer M.T., de Jong I.E., de Kleine R.H. (2022). Sequential hypothermic and normothermic machine perfusion enables safe transplantation of high-risk donor livers. Am. J. Transplant..

[11] Gilbo N., Jacquemin M., Nasralla D., Lazzaro S., Libbrecht L., Lavend’homme R., Peerlinck K., Ploeg R.J., Friend P.J., Pirenne J. (2022). Coagulation Factors Accumulate During Normothermic Liver Machine Perfusion Regardless of Donor Type and Severity of Ischemic Injury. Transplantation.

[12] Weissenbacher A., Bogensperger C., Oberhuber R., Meszaros A., Gasteiger S., Ulmer H., Berchtold V., Krendl F.J., Fodor M., Messner F. (2022). Perfusate Enzymes and Platelets Indicate Early Allograft Dysfunction After Transplantation of Normothermically Preserved Livers. Transplantation.

[13] Quintini C., Del Prete L., Simioni A., Del Angel L., Uso T.D., D’amico G., Hashimoto K., Aucejo F., Fujiki M., Eghtesad B. (2022). Transplantation of declined livers after normothermic perfusion. Surgery.

[14] Reiling J., Butler N., Simpson A., Hodgkinson P., Campbell C., Lockwood D., Bridle K., Santrampurwala N., Britton L., Crawford D. (2020). Assessment and Transplantation of Orphan Donor Livers: A Back-to-Base Approach to Normothermic Machine Perfusion. Liver Transplant..

[15] Mergental H., Perera M.T., Laing R.W., Muiesan P., Isaac J.R., Smith A., Stephenson B., Cilliers H., Neil D., Hübscher S. (2016). Transplantation of Declined Liver Allografts Following Normothermic Ex-Situ Evaluation. Am. J. Transplant..

[16] Mergental H., Laing R.W., Kirkham A.J., Perera M.T.P.R., Boteon Y.L., Attard J., Barton D., Curbishley S., Wilkhu M., Neil D.A.H. (2020). Transplantation of discarded livers following viability testing with normothermic machine perfusion. Nat. Commun..

[17] Watson C.J.E., Kosmoliaptsis V., Randle L.V., Gimson A.E., Brais R., Klinck J.R., Hamed M., Hamed M., Tsyben A., Butler A.J. (2017). Normothermic Perfusion in the Assessment and Preservation of Declined Livers Before Transplantation: Hyperoxia and Vasoplegia-Important Lessons From the First 12 Cases. Transplantation.

[18] Chen Z., Wang T., Chen C., Zhao Q., Ma Y.M., Guo Y.M., Hong X.M., Yu J., Huang C., Ju W. (2022). Transplantation of Extended Criteria Donor Livers Following Continuous Normothermic Machine Perfusion Without Recooling. Transplantation.

[19] Seidita A., Longo R., Di Francesco F., Tropea A., Calamia S., Panarello G., Barbara M., Gruttadauria S. (2022). The use of normothermic machine perfusion to rescue liver allografts from expanded criteria donors. Updat. Surg..

[20] Gaurav R., Butler A.J., Kosmoliaptsis V., Mumford L., Fear C., Swift L., Fedotovs A., Upponi S., Khwaja S., Richards J. (2022). Liver Transplantation Outcomes From Controlled Circulatory Death Donors: SCS vs in situ NRP vs ex situ NMP. Ann Surg..

[21] Fodor M., Cardini B., Peter W., Weissenbacher A., Oberhuber R., Hautz T., Otarashvili G., Margreiter C., Maglione M., Resch T. (2021). Static cold storage compared with normothermic machine perfusion of the liver and effect on ischaemic-type biliary lesions after transplantation: A propensity score-matched study. Br. J. Surg..

[22] Cardini B., Oberhuber R., Fodor M., Hautz T., Margreiter C., Resch T., Scheidl S., Maglione M., Bösmüller C., Mair H. (2020). Clinical Implementation of Prolonged Liver Preservation and Monitoring Through Normothermic Machine Perfusion in Liver Transplantation. Transplantation.

[23] Guo Z., Zhao Q., Huang S., Huang C., Wang D., Yang L., Zhang J., Chen M., Wu L., Zhang Z. (2021). Ischaemia-free liver transplantation in humans: A first-in-human trial. Lancet Reg. Health-West. Pac..

[24] Zhang Z., Tang Y., Zhao Q., Wang L., Zhu C., Ju W., Wang D., Yang L., Wu L., Chen M. (2020). Association of Perfusion Characteristics and Posttransplant Liver Function in Ischemia-Free Liver Transplantation. Liver Transplant..

[25] Liu Q., Hassan A., Pezzati D., Soliman B., Lomaglio L., Grady P., Diaz L.D.A., Simioni A., Maikhor S., Etterling J. (2020). Ex Situ Liver Machine Perfusion: The Impact of Fresh Frozen Plasma. Liver Transplant..

[26] Selzner M., Goldaracena N., Echeverri J., Kaths J.M., Linares I., Selzner N., Serrick C., Marquez M., Sapisochin G., Renner E.L. (2016). Normothermic ex vivo liver perfusion using steen solution as perfusate for human liver transplantation: First North American results. Liver Transplant..

[27] Bral M., Dajani K., Leon Izquierdo D., Bigam D., Kneteman N., Ceresa C.D.L., Friend P.J., Shapiro A.M., James A. (2019). Back-to-Base Experience of Human Normothermic Ex Situ Liver Perfusion: Does the Chill Kill?. Liver Transpl..

[28] Ceresa C.D.L., Nasralla D., Watson C.J.E., Butler A.J., Coussios C.C., Crick K., Hodson L., Imber C., Jassem W., Knight S.R. (2019). Transient Cold Storage Prior to Normothermic Liver Perfusion May Facilitate Adoption of a Novel Technology. Liver Transplant..

[29] Markmann J.F., Abouljoud M.S., Ghobrial R.M., Bhati C.S., Pelletier S.J., Lu A.D., Ottmann S., Klair T., Eymard C., Roll G.R. (2022). Impact of Portable Normothermic Blood-Based Machine Perfusion on Outcomes of Liver Transplant: The OCS Liver PROTECT Randomized Clinical Trial. JAMA Surg..

[30] Nasralla D., Coussios C.C., Mergental H., Akhtar M.Z., Butler A.J., Ceresa C.D.L., Chiocchia V., Dutton S.J., García-Valdecasas J.C., Heaton N. (2018). A randomized trial of normothermic preservation in liver transplantation. Nature.

[31] Ghinolfi D., Rreka E., De Tata V., Franzini M., Pezzati D., Fierabracci V., Masini M., Cacciatoinsilla A., Bindi M.L., Marselli L. (2019). Pilot, Open, Randomized, Prospective Trial for Normothermic Machine Perfusion Evaluation in Liver Transplantation From Older Donors. Liver Transplant..

[32] MacConmara M., Hanish S.I., Hwang C.S., De Gregorio L., Desai D.M., Feizpour C.A., Tanriover B., Markmann J.F., Zeh H., Vagefi P.A. (2020). Making Every Liver Count: Increased Transplant Yield of Donor Livers Through Normothermic Machine Perfusion. Ann. Surg..

[33] Bral M., Gala-Lopez B., Bigam D., Kneteman N., Malcolm A., Livingstone S., Andres A., Emamaullee J., Russell L., Coussios C. (2017). Preliminary Single-Center Canadian Experience of Human Normothermic Ex Vivo Liver Perfusion: Results of a Clinical Trial. Am. J. Transplant..

[34] Ravikumar R., Jassem W., Mergental H., Heaton N., Mirza D., Perera M.T.P.R., Quaglia A., Holroyd D., Vogel T., Coussios C.C. (2016). Liver Transplantation After Ex Vivo Normothermic Machine Preservation: A Phase 1 (First-in-Man) Clinical Trial. Am. J. Transplant..

[35] Jassem W., Xystrakis E., Ghnewa Y.G., Yuksel M., Pop O., Martinez-Llordella M., Jabri Y., Huang X., Lozano J.J., Quaglia A. (2019). Normothermic Machine Perfusion (NMP) Inhibits Proinflammatory Responses in the Liver and Promotes Regeneration. Hepatology.

[36] Ionescu M.-I., Tillakaratne S., Hodson J., Gunson B., Nasralla D., Boteon A.P.C.D.S., Sermon K., Mergental H., Isaac J.R., Roberts J.K. (2019). Normothermic Machine Perfusion Enhances Intraoperative Hepatocellular Synthetic Capacity: A Propensity Score-matched Analysis. Transplantation.

[37] Liu Q., Del Prete L., Hassan A., Pezzati D., Bilancini M., D’amico G., Uso T.D., Hashimoto K., Aucejo F., Fujiki M. (2022). Two pumps or one pump? A comparison of human liver normothermic machine perfusion devices for transplantation. Artif. Organs.

[38] Watson C.J.E., Kosmoliaptsis V., Randle L.V., Russell N.K., Griffiths W.J.H., Davies S., Mergental H., Butler A.J. (2016). Preimplant Normothermic Liver Perfusion of a Suboptimal Liver Donated After Circulatory Death. Am. J. Transplant..

[39] Olthoff K.M., Kulik L., Samstein B., Kaminski M., Abecassis M., Emond J., Shaked A., Christie J.D. (2010). Validation of a current definition of early allograft dysfunction in liver transplant recipients and analysis of risk factors. Liver Transplant..

[40] Glanemann M., Langrehr J.M., Stange B.J., Neumann U., Settmacher U., Steinmüller T., Neuhaus P. (2003). Clinical Implications of Hepatic Preservation Injury After Adult Liver Transplantation. Am. J. Transplant..

[41] Eisenbach C., Encke J., Merle U., Gotthardt D., Weiss K., Schneider L., Latanowicz S., Spiegel M., Engelmann G., Stremmel W. (2009). An Early Increase in Gamma Glutamyltranspeptidase And Low Aspartate Aminotransferase Peak Values Are Associated With Superior Outcomes After Orthotopic Liver Transplantation. Transplant. Proc..

[42] Vogel T., Brockmann J.G., Quaglia A., Morovat A., Jassem W., Heaton N.D., Coussios C.C., Friend P.J. (2017). The 24-hour normothermic machine perfusion of discarded human liver grafts. Liver Transplant..

[43] Mergental H., Stephenson B.T.F., Laing R.W., Kirkham A.J., Neil D.A.H., Wallace L.L., Boteon Y.L., Widmer J., Bhogal R.H., Perera M.T.P.R. (2018). Development of Clinical Criteria for Functional Assessment to Predict Primary Nonfunction of High-Risk Livers Using Normothermic Machine Perfusion. Liver Transplant..

[44] Eshmuminov D., Becker D., Bautista Borrego L., Hefti M., Schuler M.J., Hagedorn C., Muller X., Mueller M., Onder C., Graf R. (2020). An integrated perfusion machine preserves injured human livers for 1 week. Nat. Biotechnol..

[45] Lau N., Ly M., Dennis C., Liu K., Kench J., Crawford M., Pulitano C. (2022). Long-term normothermic perfusion of human livers for longer than 12 days. Artif. Organs.

[46] Lau N.S., Ly M., Jacques A., Ewenson K., Mestrovic N., Almoflihi A., Koutalistras N., Liu K., Kench J., McCaughan G. (2021). Prolonged Ex Vivo Normothermic Perfusion of a Split Liver: An Innovative Approach to Increase the Number of Available Grafts. Transplant. Direct.

[47] Lau N.-S., Ly M., Dennis C., Ewenson K., Ly H., Huang J.L., Cabanes-Creus M., Chanda S., Wang C., Lisowski L. (2023). Liver splitting during normothermic machine perfusion: A novel method to combine the advantages of both in-situ and ex-vivo techniques. HPB.

[48] Clavien P.-A., Dutkowski P., Mueller M., Eshmuminov D., Borrego L.B., Weber A., Muellhaupt B., Da Silva R.X.S., Burg B.R., von Rohr P.R. (2022). Transplantation of a human liver following 3 days of ex situ normothermic preservation. Nat. Biotechnol..

[49] Norton W.G., Pearson R., Devlin J., Nicholson M.L., Hosgood S.A. (2022). Normothermic Machine Perfusion in Renal Transplantation. Curr. Transplant. Rep..

[50] Warnecke G., Van Raemdonck D., A Smith M., Massard G., Kukreja J., Rea F., Loor G., De Robertis F., Nagendran J., Dhital K.K. (2018). Normothermic ex-vivo preservation with the portable Organ Care System Lung device for bilateral lung transplantation (INSPIRE): A randomised, open-label, non-inferiority, phase 3 study. Lancet Respir. Med..

[51] Slama A., Schillab L., Barta M., Benedek A., Mitterbauer A., Hoetzenecker K., Taghavi S., Lang G., Matilla J., Ankersmit H. (2017). Standard donor lung procurement with normothermic ex vivo lung perfusion: A prospective randomized clinical trial. J. Heart Lung Transplant..

[52] Mazilescu L.I., Parmentier C., Kalimuthu S.N., Ganesh S., Kawamura M., Goto T., Noguchi Y., Selzner M., Reichman T.W. (2022). Normothermic ex situ pancreas perfusion for the preservation of porcine pancreas grafts. Am. J. Transplant..

